# Professional burnout and increased workload during covid-19 in higher education teachers in monteria - colombia

**DOI:** 10.1192/j.eurpsy.2021.718

**Published:** 2021-08-13

**Authors:** E.P. Ruiz Gonzalez, A.M. Romero Otalvaro, M.N. Muñoz Argel, A. Uribe Urzola

**Affiliations:** Psychology, Universidad Pontificia Bolivariana, Monteria, Colombia

**Keywords:** workload, burnout, teachers, COVID-19

## Abstract

**Introduction:**

Faced with the global health emergency, a product of Covid-19, the educational system was forced to change its dynamics, assuming new challenges and adapting to virtual environments (Sierra, López, Azar & Trevethan, 2020). In this sense, the teaching task from home supposes an increase in the hours dedicated to their work, since they have seen the need to be trained in digital platforms to be able to respond to the changes derived from confinement; which can quietly generate professional wear and tear.

**Objectives:**

Analyze the relationship between professional burnout and increased workload on teachers

**Methods:**

A cross-sectional study of correlational scope was carried out in 60 (n = 60) teachers, working actively at a higher education institution. A sociodemographic scale was designed to identify the hours dedicated to work before and during confinement and to evaluate professional burnout, the adaptation of the MBI instrument for the Colombian population was used (Barbato, Córdoba, González, Martínez & Tamayo, 2011)

**Results:**

A 50% increase in the workload of teachers was observed during confinement, besides, a statistically significant correlation between professional burnout and the increase in hours dedicated to work (Table 1)

**Figure:**
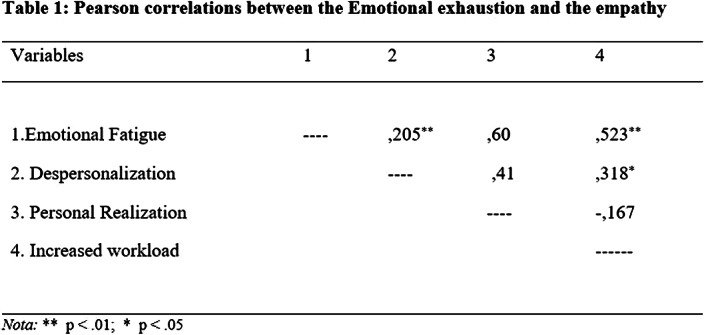

**Conclusions:**

It was possible to conclude that the significant increase in the workload in teachers correlates positively with levels of emotional exhaustion and depersonalization; however, no correlations were observed between workload and personal achievement of higher education teachers.

